# Roles of small peptides encoded by non-coding RNAs in tumor invasion and migration

**DOI:** 10.3389/fphar.2024.1442196

**Published:** 2024-09-16

**Authors:** Jie Liu, Xiyue Chang, Laeeqa Manji, Zhijie Xu, Wan’an Xiao

**Affiliations:** ^1^ Department of Orthopedics, Shengjing Hospital of China Medical University, Shenyang, Liaoning, China; ^2^ Department of Epidemiology, School of Public Health, China Medical University, Shenyang, Liaoning, China

**Keywords:** non-coding RNA, tumor, small peptide, invasion, migration

## Abstract

Non-coding RNAs (ncRNAs), which are usually considered not to encode proteins, are widely involved in important activities including signal transduction and cell proliferation. However, recent studies have shown that small peptides encoded by ncRNAs (SPENs) have important roles in the development of malignant tumors. Some SPENs participate in the regulation of skeleton reorganization, intercellular adhesion, signaling and other processes of tumor cells, with effects on the invasive and migratory abilities of the cells. Therefore, SPENs have potential applications as therapeutic targets and biomarkers of malignant tumors. Invasion and migration of malignant tumor cells are the main reasons for poor prognosis of cancer patients and represent the most challenging aspects of treatment of malignant tumors. Currently, the main treatments for tumors include surgery, radiotherapy, targeted drug therapy. Surgery, however, is reserved for early stages of cancer and carries risks and costs. Radiotherapy and targeted therapy have serious side effects. This review describes the mechanisms of SPENs and their roles in tumor invasion and migration, with the aim of providing new targets for tumor diagnosis and treatment.

## 1 Introduction

Malignant tumors, which are associated with high morbidity and mortality, represent a global public health challenge (Liu et al., 2024). Owing to the difficulty of early diagnosis, some tumors are found in the middle to late stages of the disease, with serious effects on patient survival and prognosis. Therefore, in-depth research is needed to identify biomarkers involved in the regulation of malignant transformation to better predict the progression of malignant tumors. These could have applications in both clinical diagnosis and targeted adjuvant therapy to improve the overall survival of patients (Li et al., 2022).

Only about 2% of the genes in the human genome are coding genes; the vast majority of transcripts lack coding open reading frames and are regarded as non-coding RNAs (ncRNAs); these include microRNAs, circular RNAs, and long ncRNAs (Miano et al., 2021; Panni et al., 2020; Wang J. et al., 2019; Wang Y. et al., 2019). However, owing to technological advances, circular RNAs and lncRNAs have been found to have short open reading frames that can be translated into small peptides of about 100 amino acids (aa) in length (Gao et al., 2024; Zhou et al., 2024). These small peptides, in turn, have been shown to have various biological functions, which are listed in the corresponding databases (Andrews and Rothnagel, 2014; Dragomir et al., 2020). Many ncRNAs are similar to coding RNAs (messenger RNAs, mRNAs) and are also transcribed by RNA polymerase II via the processes of polyadenylation, 5′-end capping and RNA splicing (Chen, 2016). In addition, deep sequencing of ribosomal profiles has shown that many transcripts of ncRNAs bind to the ribosome (Gelsinger et al., 2020), suggesting that ncRNAs may be able to encode proteins in the same way as mRNAs.

Small peptides encoded by ncRNAs (SPENs) have important roles in organisms. In recent years, studies published in *Science*, *Nature*, *Cell*, *Molecular Cancer* and other journals have reported that some SPENs (less than 100 aa in length, e.g., sarco-endoplasmic reticulum Ca^2+^, Toddler, myoregulin, small regulatory polypeptide of amino acid response) are involved in the processes of myogenesis, embryogenesis, and tumorigenesis (Nelson et al., 2016; Matsumoto et al., 2017; Anderson et al., 2015; Wu et al., 2020) Cancer-associated small integral membrane open reading frame 1 was the first functional small peptide found to be carcinogenic; it interacts with squalene epoxidase, a key enzyme in cholesterol synthesis, to regulate the metabolic homeostasis of cancer cells. Knockdown of cancer-associated small integral membrane open reading frame 1 led to a reduction in the proliferation of breast cancer cells (Polycarpou-Schwarz et al., 2018). In another study, HOXB-AS3, a 53-aa conserved small peptide encoded by the lncRNA HOXB-AS3, was shown to inhibit the growth of colon cancer, and its deletion was identified as a key oncogenic factor in colon cancer metabolism (Huang et al., 2017). In addition, the 59-aa small peptide SMIM30, encoded by lncRNA LINC00998, has been reported to promote hepatocellular carcinoma development by regulating cell proliferation and migration (Pang et al., 2020) These studies indicate that SPENs participate in tumorigenesis and development of tumors through complex mechanisms. This enriches our knowledge of tumor regulatory molecules, providing new directions for tumor research with potential clinical applications (Zhu et al., 2020).

Invasion and migration are central processes in tumor biology. They are critical to tumor development owing to their role in metastasis and are major causes of poor patient prognosis and cancer-related mortality (Ahmad et al., 2023). This review focuses on the mechanisms of SPENs and their roles in tumor invasion and migration, aiming to provide new strategies for clinical diagnosis and treatment of various cancers.

## 2 Invasion and migration

### 2.1 Overview of tumor cell invasion and migration

Invasion is the process by which tumor cells break out of their original location of growth and invade surrounding normal tissues. The cancer cells use a variety of mechanisms to destroy the structure of the surrounding tissue so that they can spread and metastasize to other sites. Migration, on the other hand, refers to the ability of tumor cells to spread to other parts of the body through the blood or lymphatic system. This process can be roughly divided into the following three steps. First, the migration ability of cancer cells is enhanced after they are detached from the tumor, enabling them to further destroy the extracellular matrix (ECM) and basement membrane and form an invasive growth in normal tissue (Joshi et al., 2023). Second, the surviving cancer cells escape from the wall of the blood vessels, grow and proliferate in suitable tissues and organs, and form a metastatic cancerous focus. Last, a network of neovascularization is formed inside the metastatic focus, which promotes proliferation of the cancerous cells and enables a new round of metastasis (Spano et al., 2012). Invasion and migration processes have crucial roles in the development and progression of tumors. Abnormally active cell migration and invasion mechanisms lead to the formation of abnormal structures around the tumor, resulting in the development of invasive cancers. Therefore, elucidating the mechanisms of these processes can provide a better understanding the mechanisms of cancer development and enable the development more effective therapeutic strategies to improve patient survival and quality of life.

### 2.2 Roles of invasion and migration in tumorigenesis and development of tumors

Invasion and migration are important aspects of tumor dissemination, and together they constitute the process of tumor metastasis (van Zijl et al., 2011). This process can cause extensive harm and also represents a challenge in cancer treatment, as widespread metastasis often means that advanced cancer cannot be surgically eradicated (Izdebska et al., 2023).

Invasion and migration are central processes in tumor biology (Wu et al., 2021). Invasion involves the degradation and penetration of tumor cells into surrounding tissues, whereas migration involves the movement of tumor cells and colonization of new sites. The two processes are usually driven by complex interactions between tumor cells and the surrounding environment. The molecular mechanisms of invasion and migration are complex and varied. Cancer cells gain motility by remodeling of their tight cell–cell and cell–matrix adhesions, which allows them to leave the primary tumor and invade surrounding tissues (Valastyan and Weinberg, 2011; Christofori, 2006; Lim et al., 2017). Then, the cancer cells secrete a variety of enzymes to degrade the matrix of surrounding tissues. These enzymes break down the ECM and basement membrane stroma, thereby providing a pathway for cancer cells to spread and metastasize (Mondal et al., 2020). In addition, cancer cells release signaling factors, which promote invasion and metastasis by activating signals associated with these processes (Hashemi Goradel et al., 2019; Todd and Johnson, 2020) These changes allow tumor cells to escape the primary site and form new tumor foci at distant sites.

## 3 SPENs

For a long time, RNAs have been divided into two main categories: mRNAs and ncRNAs (Yasuda and Hayashizaki, 2008). mRNAs act as carriers of genetic information and direct protein synthesis, whereas ncRNAs were once called the “dark matter of the genome” because they were thought to not directly code for proteins. However, recent studies have revealed that some ncRNAs can encode small peptides. This discovery has greatly expanded our understanding of RNA function and opened a new chapter in biological research.

Small peptides encoded by ncRNAs are synthesized through different mechanisms from those associated with classical mRNA-encoded proteins. These comprise ribosome-independent mechanisms, involving the regulation of tRNA half-life, editing, and modification; and ribosome-dependent mechanisms, in which the open reading frames of the ncRNAs are recognized by the ribosome and translated into small peptides.

SPENs have a small molecular weight, usually between 10 and 100 aa. Despite their small molecular weight, these peptides have important regulatory functions in organisms, including signal transduction, protein interactions, and regulation of gene expression (Dong et al., 2023). In addition, SPENs usually have high specificity and sensitivity and function under specific physiological or pathological conditions. SPENs perform a wide variety of functions in organisms, participating in cell signal transduction, cell proliferation, differentiation and apoptosis. SPENs can also interact with specific proteins to change their activity or localization, as well as acting as molecular chaperones and participating in the assembly and regulation of other biomolecules. SPENs also have important roles in the onset and development of cancers.

## 4 Mechanisms of SPENs in tumor invasion and migration

**FIGURE 1:**
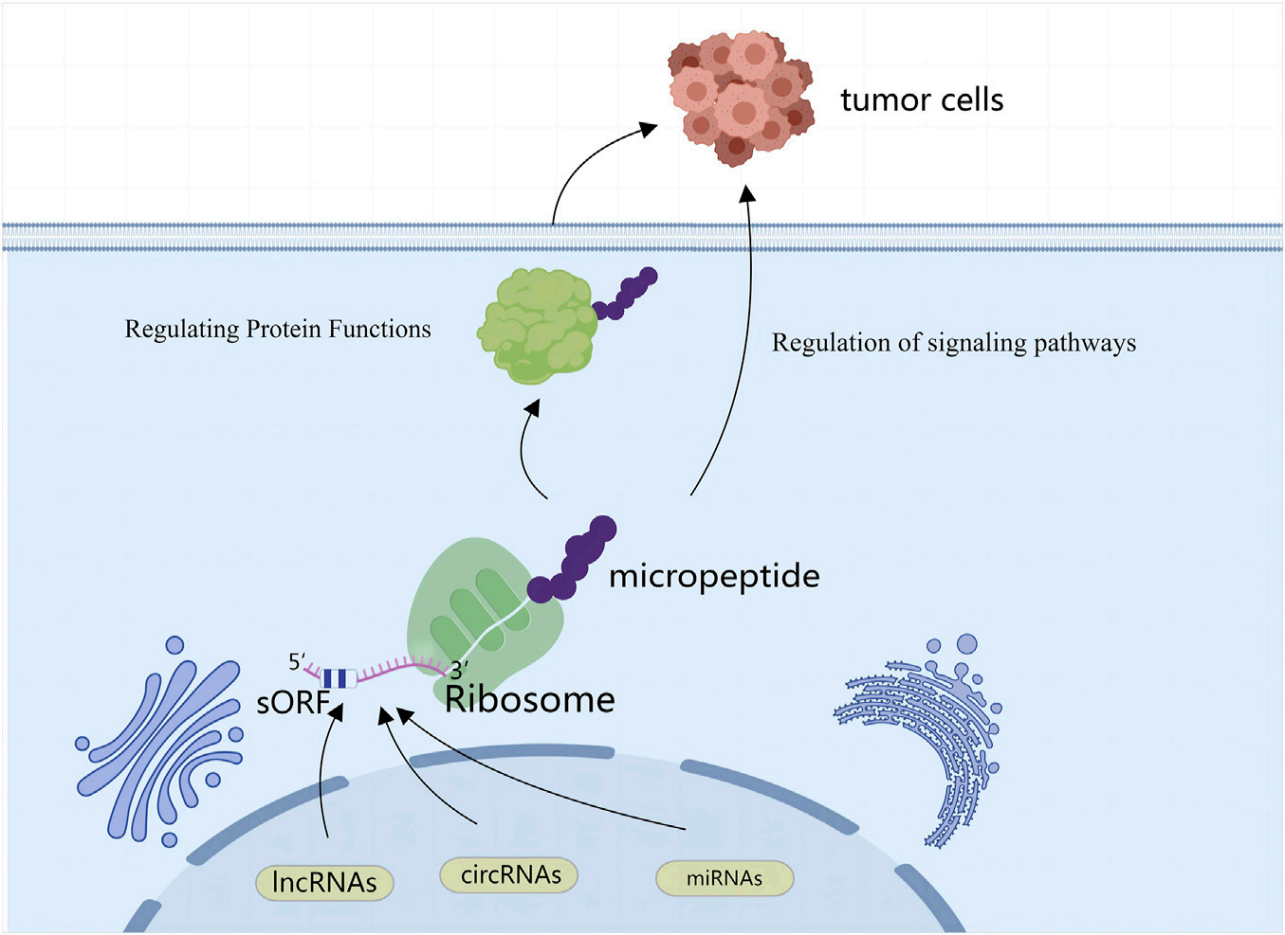
SPENs refer to small peptides encoded by ncRNAs (lncRNAs, circRNAs, miRNAs). Some ncRNAs can generate small peptides through unconventional translation mechanisms, and these peptides influence behaviors of cancer cells by regulating protein functions and participating in signaling pathways.

**FIGURE 2:**
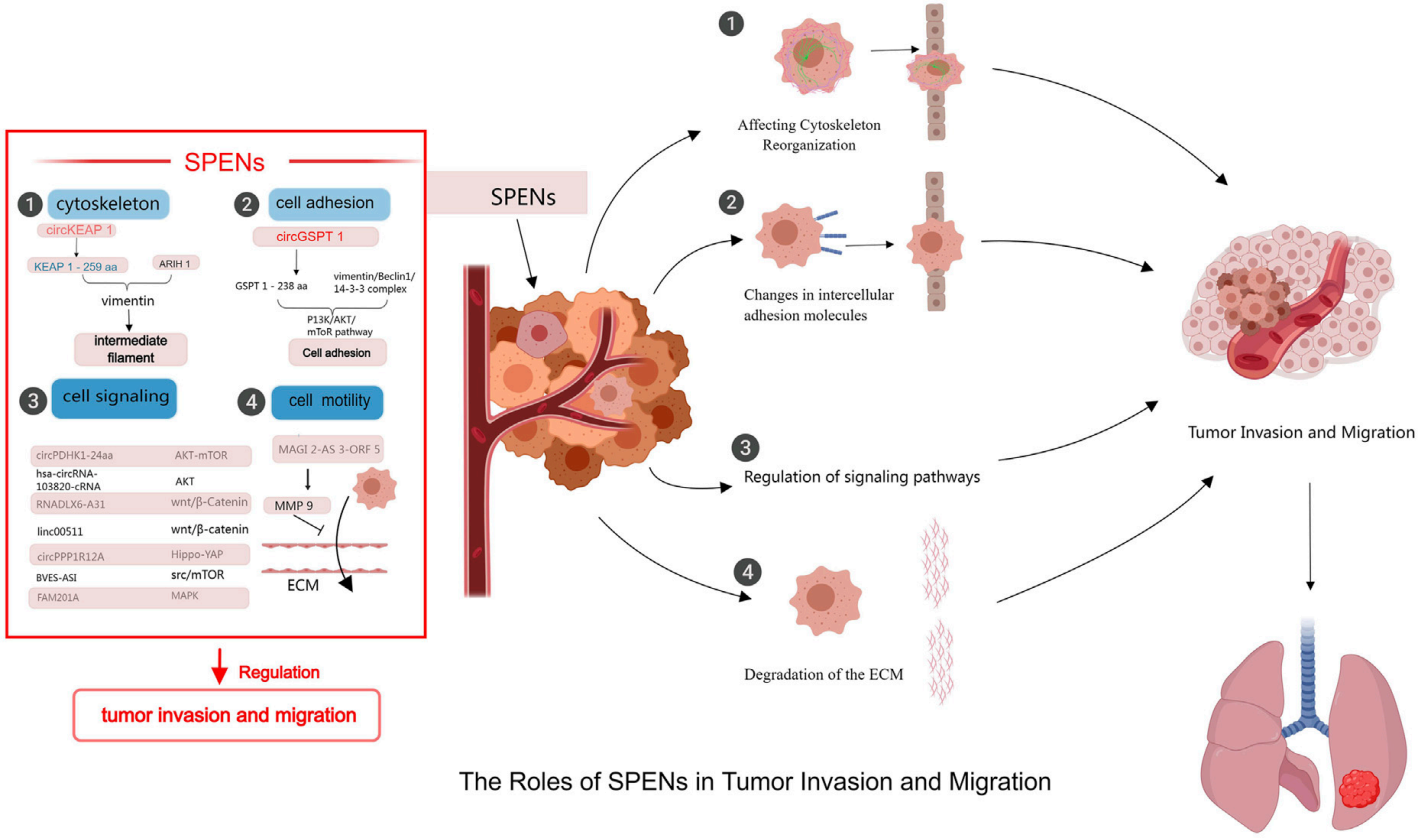
Mechanisms of SPENs on tumor invasion and migration. SPENs play significant roles in tumor invasion and metastasis by regulating protein functions, participating in signal transduction, affecting cytoskeleton reorganization, and influencing the extracellular matrix.

Although relatively little research has focused on the roles of SPENs in tumor biology, some studies have provided insight into their functions. SPENs play important parts in cancer cell invasion and migration through a variety of mechanisms (Figure 1). For example, they can regulate protein functions and activities by binding to specific proteins and forming complexes. These proteins may be involved in processes such as cell adhesion and cell signaling, which have crucial roles in tumor cell invasion and migration; thus the regulation of these processes by SPENs may directly affect the migratory and invasive behavior of tumor cells. In addition, SPENs can influence the reorganization and dynamics of the cytoskeleton, an important structure within the cell that maintains cell morphology and motility. Through effects on the assembly and stability of the cytoskeleton, small peptides proximally alter cell morphology and motility, thereby playing a key part in tumor invasion and migration (Hu et al., 2022; Zhang Y. et al., 2024; Ye et al., 2023) (Figure 2).

In recent years, studies of SPENs have confirmed that several ncRNAs can encode small peptides and regulate various malignant tumor phenotypes (Polycarpou-Schwarz et al., 2018). Mechanisms of tumor-associated functional peptides encoded by ncRNAs in invasion and migration have been reported in the following tumor types: gastric cancer, intestinal cancer, clear cell renal cell carcinoma, osteosarcoma, neuroblastoma, lung cancer, and breast cancer (Table 1).

**TABLE 1:** Mechanisms of SPENs in tumor invasion and migration.

Tumor types		ncRNAs	SPENs	Mechanisms of SPENs	Published Journals	References information
Gastric cancer		CircGSPT1	GSPT1-238aa	Waveform protein/Beclin 1/14-3-3 complex interacts with GSPT1-238aa and regulates autophagy in gastric cancer cells via the PI3K/AKT/mTOR signaling pathway	*Cancer Letters*	Hu et al. (2022)
Colorectal cancer	Colon	circPPP1R12A	circPPP1R12A-73aa	Promotion of colon cancer growth and metastasis through activation of the Hippo-YAP signaling pathway	*Molecular Cancer*	Zheng et al. (2019)
	Colorectal cancer	lncRNA BVES-AS1	BVES-AS1-201-50aa	BVES-AS1-201-50aa enhances activation of the Src/mTOR pathway in colorectal cancer cells and promotes cell migration and invasion of colorectal cancer cells	*PLoS One*	Zheng et al. (2023)
Clear cell renal cell carcinoma		circPDHK1	PDHK1-241aa	Inhibition of AKT dephosphorylation and activation of the AKT-mTOR signaling pathway through interaction with PPP1CA promotes clear cell renal cell carcinoma progression	*Molecular Cancer*	Huang et al. (2024)
Osteosarcoma		circKEAP1	KEAP1-259aa	Binds to wave proteins in the cytoplasm to promote wave protein proteasomal degradation through interaction with the E3 ligase ARIH1	*Journal of Experimental and Clinical Cancer Research*	Zhang et al. (2024a)
Neuroblastoma		FAM201A	NBASP	Neuroblastoma- associated small protein interacts with FABP5 via the ubiquitin proteasome pathway and reduces FABP5 expression, thereby inhibiting neuroblastoma through the MAPK signaling pathway	*Communications Biology*	Ye et al. (2023)
		circSHPRH	SHPRH-146aa	Apoptosis is induced mainly by regulating key apoptotic proteins caspase-3 and Bcl-2; in addition, circ-SHPRH peptide-RUNX 1 interaction enhances expression of NFKBIA in neuroblastoma, which plays an important part in inhibiting the malignant progression of neuroblastoma	*PeerJ*	Gao et al. (2024)
Lung cancer	Uncategorized lung cancer	hsa_circRNA_103820	hsa_circRNA_103820 188-aa	Suppression of malignant progression of lung cancer cells by inhibiting the AKT pathway	*Chemical Biology and Drug Design*	Zhou et al. (2024)
	Non-small-cell lung cancer	DLX6-AS1	SMIM30	Exogenous overexpression of SMIM30 promotes non-small-cell lung cancer growth through activation of the Wnt/β-catenin pathway	*Critical Reviews in Eukaryotic Gene Expression*	Xu et al. (2022)
		circβ-catenin	circβ-catenin-β370aa	Inhibition of β-catenin degradation by binding GSK3β promotes a malignant phenotype in non-small-cell lung cancer cells	*Journal of Clinical Laboratory Analysis*	Zhao et al. (2021)
	Adenocarcinoma of the lungs	LINC 00954	LINC 00954-ORF	Enhances sensitivity to PEM (anticancer drug) and inhibits A549/PEM cell growth	*Amino Acids*	Han et al. (2024)
Lymphoma		MAGI2-AS3	magi2-as3-orf5	Regulation of BRCA cell migration by ECM-related proteins	*Molecular Biotechnology*	Zhang et al. (2024b)

### 4.1 Changes in intercellular adhesion molecules

In normal tissues, cells adhere to each other, forming an interdependent system that promotes their survival. By contrast, cancer cells weaken intercellular adhesion and enhance cell migration by altering the expression of intercellular adhesion molecules, such as those involved in epithelial–mesenchymal transition (EMT) (Lee et al., 2017). EMT is the phenotypic transformation of epithelial cells to acquire mesenchymal features, a crucial process in tumor cell migration (Pantia et al., 2023). Waveform protein, an important marker of EMT, can cause tumor cells to lose adhesion and disrupt their tight junction structure, change the structure of the cytoskeleton, and increase the invasiveness and distant metastasis of tumor cells (Strouhalova et al., 2020; Ohara et al., 2020). Hu et al., (2022) demonstrated that a novel tumor suppressor protein, GSPT1-238aa, encoded by circGSPT1, interacts with the waveform protein/Beclin 1/14-3-3 complex and regulates autophagy in gastric cancer cells through the PI3K/AKT/mTOR signaling pathway. Zhang Y. et al., (2024) showed that KEAP1-259aa, a small peptide encoded by circKEAP1, binds to waveform proteins in the cytoplasm, where it promotes the proteasomal degradation of waveform proteins through interaction with ARIH1, an E3 ligase. In addition to mediating cell adhesion, cell adhesion molecules act as tumor suppressors, limiting tumor growth and migration through contact inhibition. Weakening of cell adhesion allows tumor cells to gain motility and invasiveness, escape the original tumor site, and metastasize to distant organs. Important adhesion molecules include integrin, cadherin, selectin, the immunoglobulin superfamily (e.g., intercellular adhesion molecule-1, neural cell adhesion molecule, vascular cell adhesion molecule, carcinoembryonic antigen, and deleted in colon cancer), CD44, and the 67 kD laminin receptor (Staff, 2001; Moh and Shen, 2009). Calcineurin family members form relatively strong calcium-dependent homotypic adhesions with neighboring cells through their cytoplasmic structural domains, thereby maintaining the stability of cell–cell adhesion. Integrin receptors are calcium-dependent heterodimers composed of noncovalently linked alpha and beta subunits. Members of the integrin family of molecules connect the ECM to the cytoskeleton and transduce signals controlling adhesion and migration in both directions. Members of the selectin family enable transient cell–cell adhesion by interacting with ligands on glycoproteins and glycolipids in the carbohydrate portion of the sialic acid-Lewis X tetrasaccharide. Members of the immunoglobulin superfamily mediate calcium-independent cell–cell adhesion through structural domains, regulate adhesion, and recognize both homophilic and heterophilic ligands (von Lersner et al., 2019).

### 4.2 Degradation of the ECM

The ECM is a fundamental core component of body tissues and organs and is essential for the existence of multicellular organisms (Cox, 2021). The ECM, which consists of collagen, polysaccharides, proteoglycans, etc., not only provides physical support for cells but also participates in essential processes such as cell growth, differentiation, migration, and signaling. ECM degradation is the pathological basis for the development of many diseases; moreover, degradation of the ECM, including the basement membrane, is a key step in tumor invasion and metastasis, because tumor cells must cross the basement membrane several times to invade surrounding tissues. The various components of the ECM are degraded by specific protein hydrolases; thus, the degradation of the entire ECM requires the synergistic action of multiple matrix hydrolases. Tumor cells facilitate their own invasion and migration by secreting enzymes that rupture the structure of the ECM, enabling them to penetrate the ECM barrier and invade surrounding tissues and blood vessels; they then travel via the circulatory system to other sites, eventually forming metastatic tumors. The proteolytic enzymes associated with ECM degradation by tumor cells can be divided into four major groups: serine proteases (e.g., plasma fibrinolytic plasminogen activator), metalloproteinases, elastases, and cysteine proteases; the first two groups have been investigated in depth. Tumor biomarkers have key roles in cancer screening, diagnosis and prognosis, detection of progression, prediction of recurrence, and evaluation of treatment efficacy (Huang, 2018). MMP9 regulates ECM degradation during tumor metastasis. Degradation and is considered a marker of cell migration and invasion. Zhang Z. et al., (2024) showed that the fluorescence intensity of MMP9 was significantly reduced by overexpression of MAGI2-AS3-ORF5, a small peptide encoded by a non-coding RNA. A transwell assay further revealed that accumulation of MAGI2-AS3-ORF5 reduced numbers of migrating breast cancer cells. In addition, it was found to regulate the migration of breast cancer cells through ECM-associated proteins.

### 4.3 Cytoskeletal alterations

The cytoskeleton, which comprises cytoplasmic cytoskeleton and nuclear cytoskeleton parts, consists of intermediate fibers, microfilaments, and microtubules, which exist in the cytoplasm and are assembled by a network of proteins. As well as its involvement in activities such as cell division, motility, material movement, and signal transduction, it plays a crucial part in maintaining the basic shape and structure of the cell. Dysfunction or abnormal expression of constituents of the cytoskeleton may thus lead to changes in cell morphology and structure, which in turn may trigger a series of physiopathological processes. Recent studies have also identified an important role of the cytoskeleton in tumor migration and invasion. Cancer cells have a high degree of plasticity, and the deformability of cells is increased by altering the structure and function of the cytoskeleton, thereby promoting cell migration and invasion. Microfilaments, the most important component of the cytoskeleton, consist of fibrinogen, actin, and microkeratin; they enable cell migration and invasion through dynamic reorganization and depolymerization. Intermediate fibers are the most stable part of the cytoskeleton and have an important role in supporting cells. Waveform protein, which is among the most important intermediate fiber proteins, provides physical scaffolding for various organelles in the cell, thereby maintaining cytoskeletal conformation and cellular morphology as well as the integrity and mobility of bridge particles (Guo et al., 2013). Zhang Y. et al. (2024) found that circKEAP1 encodes the protein KEAP1-259aa, which binds to waveform proteins and facilitates their degradation by interacting with the E3 ligase ARIH1, which in turn inhibits tumor migration.

### 4.4 Regulation of signaling pathways

Signaling pathways are intracellular information transfer systems that involve various biochemical reactions and signal transduction processes. Abnormalities of these signaling pathways may lead to cellular dysfunction. Cancer cells participate in the regulation of cell invasion and migration through activation of various signaling pathways, including the Rho GTPase family, PI3k/Akt, and MAPK pathways. A newly discovered circular RNA, circPPP1R12A, encodes a small peptide named circPPP1R12A-73aa. Zheng et al. (2019) demonstrated experimentally that circPPP1R12A-73aa promotes the migration ability and invasiveness of colon cancer through activation of the Hippo-YAP signaling pathway. Zheng et al. (2023) found that lncRNA BVES-AS1 encodes peptide BVES-AS1-201-50aa, which promotes cell migration and invasion of colorectal cancer cells by enhancing activation of the Src/mTOR pathway. Ye et al. (2023) showed that neuroblastoma-associated small protein, a small peptide encoded by FAM201A, interacts with fatty acid binding protein 5 (FABP5) through the ubiquitin proteasome pathway to reduce the expression of FABP5, thereby suppressing cellular migration and invasion through the MAPK signaling pathway and inhibiting neuroblastoma tumorigenesis. Huang et al. (2024) demonstrated that circPDHK1 encodes a novel peptide, PDHK1-241aa, which promotes invasion and metastasis of clear cell renal cell carcinoma by inhibiting AKT dephosphorylation and activating the AKT-mTOR signaling pathway through its interaction with PPP 1CA. Zhou et al., (2024) demonstrated that a 188-aa peptide encoded by hsa_circRNA_103820 inhibits cell migration and invasion by inactivating the AKT pathway in lung cancer. Xu et al., (2022) showed that the lncRNA DLX6-AS1, encoding peptide SMIM30, promotes migration and invasion of non-small-cell lung cancer cells through activation of the Wnt/β-catenin signaling pathway. Tan et al. (2023) showed that the small peptide LINC00511-133 aa is encoded by LINC00511 and promotes invasiveness of breast cancer cells by regulating expression levels of Wnt/β-catenin-pathway-associated proteins Bax, cMyc, and cyclin D1, as well as facilitating the entry of β-catenin proteins into the nucleus.

## 5 Conclusion and outlook

This article focuses on an emerging area of research: the role of SPENs in tumor invasion and migration. We review the mechanisms by which SPENs act in tumor invasion and migration, highlighting the previously unrecognized complexity of ncRNAs. It is an exciting issue to address why discove the role of SPENs in tumor invasion and migration. Invasion and migration are key processes in tumor metastasis, which is of clinical significance as the vast majority of cancer patients die from metastatic rather than primary tumors. In-depth studies of the mechanisms underlying these processes are thus conducive to the development of diagnostic and therapeutic strategies for cancer. SPENs, which represent a newly discovered function of RNAs, both enrich existing protein libraries and provide new directions for protein-related research; they also opens up new avenues for cancer treatment. Research into the coding properties of ncRNAs is ongoing; however, the detection and identification of small peptides remains challenging owing to the immaturity of experimental techniques. In addition, many studies have failed to distinguish between the functions of SPENs and those of ncRNAs themselves; this should be considered in the future. Moreover, although some SPENs have been shown to have functional features, their mechanisms of action are not yet clear; challenges remain regarding how to verify their activities and functions. Therefore, further studies are needed. However, despite our current limited understanding in this field, we expect more breakthroughs and advances as research continues and the technology develops. SPENs have the potential to play important parts in drug development and in the diagnosis and treatment of various diseases, which will be beneficial to human health and quality of life.

## Abbreviations

ncRNA, Non-coding RNAs; SPENs, small peptides encoded by non-coding RNAs; aa, amino acids; mRNA, messenger RNAs; ECM, extracellular matrix; EMT, epithelial-mesenchymal transition; FABP5, fatty acid binding protein 5.
