# Supplementary material for: Roles of small peptides encoded by non-coding RNAs in tumor invasion and migration
Source: Front Pharmacol. 2024 Sep 16;15:1442196. doi: 10.3389/fphar.2024.1442196 (PMC11439703; doi:10.3389/fphar.2024.1442196)
Supplement: Supplementary file 1 [file Table1.DOCX]

**Table 1.** Mechanisms of SPENs in tumor invasion and migration.

| Tumor types | | ncRNAs | SPENs | Mechanisms of SPENs | Published Journals | References information |
| --- | --- | --- | --- | --- | --- | --- |
| Gastric cancer | | CircGSPT1 | GSPT1-238aa | Waveform protein/Beclin 1/14-3-3 complex interacts with GSPT1-238aa and regulates autophagy in gastric cancer cells via the PI3K/AKT/mTOR signaling pathway | *Cancer Letters* | ([Hu et al., 2022](#Hu2022)) |
| Colorectal cancer | Colon | circPPP1R12A | circPPP1R12A-73aa | Promotion of colon cancer growth and metastasis through activation of the Hippo-YAP signaling pathway | *Molecular Cancer* | ([Zheng et al., 2019](#Zheng2019)) |
|  | Colorectal cancer | lncRNA BVES-AS1 | BVES-AS1-201-50aa | BVES-AS1-201-50aa enhances activation of the Src/mTOR pathway in colorectal cancer cells and promotes cell migration and invasion of colorectal cancer cells | *PLoS One* | ([Zheng et al., 2023](#Zheng2023)) |
| Clear cell renal cell carcinoma | | circPDHK1 | PDHK1-241aa | Inhibition of AKT dephosphorylation and activation of the AKT-mTOR signaling pathway through interaction with PPP1CA promotes clear cell renal cell carcinoma progression | *Molecular Cancer* | ([Huang et al., 2024](#Huang2024)) |
| Osteosarcoma | | circKEAP1 | KEAP1-259aa | Binds to wave proteins in the cytoplasm to promote wave protein proteasomal degradation through interaction with the E3 ligase ARIH1 | *Journal of Experimental & Clinical Cancer Research* | ([Zhang et al., 2024a](#Zhang2024a)) |
| Neuroblastoma | | FAM201A | NBASP | Neuroblastoma- associated small protein interacts with FABP5 via the ubiquitin proteasome pathway and reduces FABP5 expression, thereby inhibiting neuroblastoma through the MAPK signaling pathway | *Communications Biology* | ([Ye et al., 2023](#Ye2023)) |
|  |  | circSHPRH | SHPRH-146aa | Apoptosis is induced mainly by regulating key apoptotic proteins caspase-3 and Bcl-2; in addition, circ-SHPRH peptide-RUNX 1 interaction enhances expression of NFKBIA in neuroblastoma, which plays an important part in inhibiting the malignant progression of neuroblastoma | *PeerJ* | ([Gao et al., 2024](#Gao2024)) |
| Lung cancer | Uncategorized lung cancer | hsa_circRNA_103820 | hsa_circRNA_103820 188-aa | Suppression of malignant progression of lung cancer cells by inhibiting the AKT pathway | *Chemical Biology & Drug Design* | ([Zhou et al., 2024](#Zhou2024)) |
|  | Non-small-cell lung cancer | DLX6-AS1 | SMIM30 | Exogenous overexpression of SMIM30 promotes non-small-cell lung cancer growth through activation of the Wnt/β-catenin pathway | *Critical Reviews in Eukaryotic Gene Expression* | ([Xu et al., 2022](#Xu2022)) |
|  |  | circβ-catenin | circβ-catenin-β370aa | Inhibition of β-catenin degradation by binding GSK3β promotes a malignant phenotype in non-small-cell lung cancer cells | *Journal of Clinical Laboratory Analysis* | ([Zhao et al., 2021](#Zhao2021)) |
|  | Adenocarcinoma of the lungs | LINC 00954 | LINC 00954-ORF | Enhances sensitivity to PEM (anticancer drug) and inhibits A549/PEM cell growth | *Amino Acids* | ([Han et al., 2024](#Han2024)) |
| Lymphoma | | MAGI2‑AS3 | magi2-as3-orf5 | Regulation of BRCA cell migration by ECM-related proteins | *Molecular Biotechnology* | ([Zhang et al., 2024b](#Zhang2024b)) |
